# Harnessing innate lung anti-cancer effector functions with a novel bacterial-derived immunotherapy

**DOI:** 10.1080/2162402X.2017.1398875

**Published:** 2017-11-27

**Authors:** Mark Bazett, Amanda M. Costa, Momir Bosiljcic, Rebecca M. Anderson, Matthew P. Alexander, Stephanie W. Y. Wong, Salim Dhanji, Jenny MH Chen, Jim Pankovich, Stephen Lam, Simon Sutcliffe, Hal Gunn, Shirin Kalyan, David W. Mullins

**Affiliations:** a Department of Microbiology and Immunology, Geisel School of Medicine at Dartmouth, Hanover, NH, USA; b Department of Medical Education, Geisel School of Medicine at Dartmouth, Hanover, NH, USA; c Qu Biologics Inc., Vancouver, BC, V5 T 4T5, Canada; d Department of Medicine, Division of Endocrinology, University of British Columbia, Vancouver, BC, V5Z 1M9, Canada; e Department of Microbiology and Immunology, University of British Columbia, Vancouver, BC, Canada; f BC Cancer Research Center, Vancouver, BC, Canada

**Keywords:** immunotherapy, innate immunity, *Klebsiella*, lung cancer, NK cells, NKG2D, NSCLC

## Abstract

Acute infection is known to induce strong anti-tumor immune responses, but clinical translation has been hindered by the lack of an effective strategy to safely and consistently provoke a therapeutic response. These limitations are overcome with a novel treatment approach involving repeated subcutaneous delivery of a *Klebsiella*-derived investigational immunotherapeutic, QBKPN. In preclinical models of lung cancer, QBKPN administration consistently showed anti-cancer efficacy, which was dependent on *Klebsiella* pre-exposure, but was independent of adaptive immunity. Rather, QBKPN induced anti-tumor innate immunity that required NK cells and NKG2D engagement. QBKPN increased NK cells and macrophages in the lungs, altered macrophage polarization, and augmented the production of cytotoxic molecules. An exploratory trial in patients with non-small cell lung cancer demonstrated QBKPN was well tolerated, safe, and induced peripheral immune changes suggestive of macrophage polarization and reduction of PD-1 and PD-L1 expression on leukocytes. These data demonstrate preclinical efficacy, and clinical safety and tolerability, for this cancer immunotherapy strategy that exploits innate anti-tumor immune mechanisms.

## Introduction

Bacteria-induced anti-cancer immune activation is the first documented form of cancer immunotherapy, dating back to 2600 BC in Egypt.^1^ This observation was more formally explored clinically in the late 1800′s, after Dr. William Coley observed that osteosarcoma patients who developed acute postoperative infections had markedly improved survival rates.^2^ Coley went on to develop Coley's Toxin, a treatment that consisting of heat-killed *Streptococcus pyogenes* and *Serratia marcescens*,^3^ which was used to treat patients with sarcomas, carcinomas, lymphomas, myelomas and melanomas. Although Dr. Coley had some remarkable success, the lack of sufficient characterization of the cellular and molecular mechanisms driving the therapeutic effect, the inability to consistently illicit the therapeutic effect, and safety issues associated with intravenous administration hampered the full exploitation of bacterial-based immunotherapy for cancer, which fell out of favor with the advent of chemotherapy and radiation.^1^ Thus, over the past 40 years, intra-vesical Bacillus Calmette-Guérin (BCG) treatment of high-risk superficial bladder cancer has remained the only approved clinical application of this class of therapeutic, although it's mechanism of action remains uncertain.^1,4^

The renaissance of cancer immunotherapy over the last several decades, though primarily focused on modulating the adaptive arm of the immune system, has led to a multitude of studies to evaluate the use of live, attenuated, or genetically modified bacterial species to colonize tumors, enhance cancer antigen presentation, increase immune cell infiltration, and enhance tumor cytotoxicity.^1,5^ Recent experimental strategies have employed attenuated strains of *Listeria monocytogenes*^6,7^ or replication-deficient *Toxoplasma gondii*,^8–10^ demonstrating anti-tumor efficacy in multiple tumor models. Experimental chronic *Staphylococcus* osteomyelitis was demonstrated to convey protective anti-tumor immune activity,^11^ in keeping with the observation that localized post-surgical infection has been correlated with increased survival in canine^12^ and human^13,14^ osteosarcoma. While these and other recent studies demonstrate the potential for bacterial immune stimulation in the treatment of cancer, these approaches rely on infection by live bacteria reaching the tumor microenvironment via intravenous or intra-tumoral administration. These approaches are limited in clinical applications to a subset of patients with accessible and/or susceptible tumors, must be administered by a health care professional in a clinical setting, and are associated with significant potential safety risks.^5^ The goal of this study was to test a bacterial-derived immunotherapeutic that was designed to be administered subcutaneously to stimulate an innate anti-cancer immune response in the lungs, without the need for live organisms or intravenous/intra-tumoral administration.

We hypothesized that subcutaneous delivery of a bacterial immunotherapy, derived from a lung pathogen, could mimic an acute infection and stimulate an effective anti-tumor immune response in the lungs. QBKPN, a novel inactivated *Klebsiella*-based investigational therapeutic that contains all the major *Klebsiella* macromolecules, has previously been tested in murine models of asthma^15^ and chronic obstructive pulmonary disease (COPD),^16^ demonstrating therapeutic efficacy. In these animal models, QBKPN administration resulted in recruitment of innate immune cells into the lungs and amelioration of disease-specific inflammation. In the present study, QBKPN administration reduced tumor burden and increased survival through induction of innate effector mechanisms in mouse models of metastatic-like lung cancer. To assess the feasibility of this treatment strategy in the clinical setting, we performed a small exploratory trial where patients with non-small cell lung cancer (NSCLC) were treated with QBKPN for 12 weeks, providing data on safety, tolerability, tumor size, and peripheral blood immunology (NCT02256852). Collectively, the results from these studies provide proof-of-principle that this cancer immunotherapy strategy, in which QBKPN is subcutaneously administered to stimulate an acute infection-like immune response, was well tolerated and led to the activation and mobilization of lung innate immune responses and anti-cancer efficacy.

## Results

### QBKPN administration reduced tumor burden in murine models of metastatic-like lung cancer

**Figure 1.**
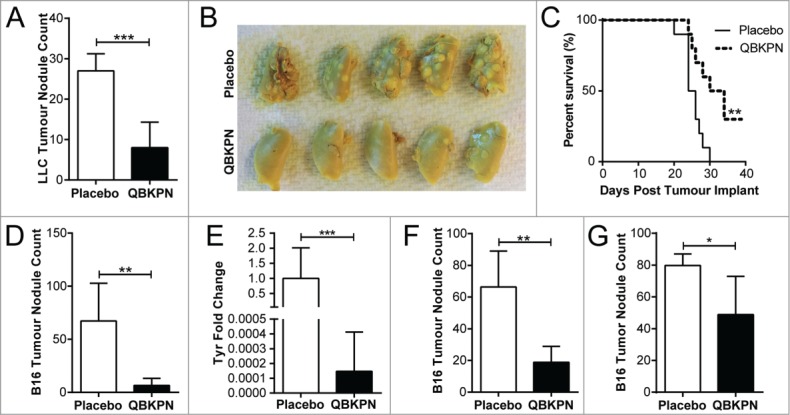
QBKPN was efficacious in models of metastatic-like lung cancers. (A) LLC left lung surface tumor nodule counts and (B) tumor visualization using Bouin's fixative, in mice 14 days after lung tumor challenge via tail vein injection (day 0). QBKPN or placebo was administered subcutaneously every other day starting 10 days before tumor challenge, until the end of the experiment. (C) Survival following LLC challenge in mice administered with placebo or QBKPN from day −10 until euthanasia. (D) B16F10 lung surface tumor nodule counts, and (E), the melanoma specific Tyrosinase (*Tyr)* gene expression in the post-caval lung lobe, on day 17 post-tumor challenge (tail vein), in mice administered with placebo or QBKPN from day −10 until day 17. (F) Day 20 B16F10 lung surface tumor nodule counts of therapeutically treated mice (every-other-day placebo or QBKPN treatment initiated 1 day post-tumor challenge). (G) Day 14 B16F10 lung surface tumor nodule counts of therapeutically treated mice with treatment starting on day 5 post-tumor implantation. For tumor nodule counts and *Tyr* expression, mean +/− SD shown. N = 4–5 mice per group. *P < 0.05; **P < 0.01; ***P < 0.001 by Student's *t*-test. For Kaplan Myer survival plots, n = 10 mice per group, ** P < 0.01 by Log-rank test.

Although innate immunity is capable of mediating significant anti-tumor effects, bacterial-induced innate effector functions are currently underutilized in the context of cancer immunotherapy. We hypothesized that repeated administration of the immunotherapy, QBKPN, would induce acute inflammation and an innate immune response in the lungs, and mediate anti-cancer efficacy in murine models of metastatic-like lung disease. In a Lewis Lung Carcinoma (LLC) lung cancer mouse model in which QBKPN was administered every second day starting 10 days before tumor inoculation until the end of the experiment, QBKPN intervention reduced tumor foci (Fig. 1A, B) and increased survival (Fig. 1C). Importantly, QBKPN efficacy was not limited to lung cancer cell lines, since QBKPN administration reduced tumor burden in the B16F10 melanoma lung metastatic model (Fig. 1D, E).^17^ Differences in tumor development were not a consequence of differential engraftment, as prophylactic QBKPN administration did not impact melanoma engraftment into the lungs, assessed two hours post-injection by qualitative RT-PCR for melanoma specific gene products (data not shown).^18^ To determine whether QBKPN could be used as a treatment strategy post tumor inoculation, therapeutic administration of QBKPN was tested in tumor-challenged mice. QBKPN treatment decreased tumor burden of B16F10 melanoma when treatment was initiated at day +1 (Fig. 1 F) or day +5 (Fig. 1G) post-tumor inoculation (by which time the tumor has engrafted^18,19^), suggesting that QBKPN treatment can induce anti-tumor immune activity in the context of a growing tumor.

### QBKPN-mediated anti-tumor efficacy required host prior exposure to Klebsiella

**Figure 2.**
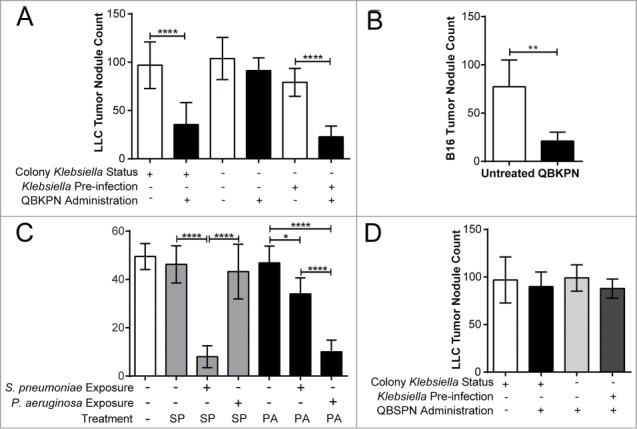
QBKPN anti-tumor efficacy required host exposure to *Klebsiella*. (A) LLC lung surface tumor nodule counts at day 18 in animals administered QBKPN or left untreated from day −10 to day 16, obtained from facilities testing positive for *Klebsiella* species or *Klebsiella*-excluded facilities. *Klebsiella* pre-infection of certain groups occurred 21 days before the beginning of the experiment (day −31). (B) Day 14 B16F10 lung surface tumor nodule count in mice pre-infected with *Klebsiella* >6 months prior to tumor challenge and administration of QBKPN (day −10 to day 12). (C) Day 18 LLC lung surface tumor nodule counts in mice pre-infected with *Streptococcus pneumoniae* or *Pseudomonas aeruginosa* (21 days before treatment, as described for *Klebsiella*) and administered a Streptococcus pneumoniae-derived immunotherapy (QBSPN) or a *Pseudomonas*-derived immunotherapy (QBPAE) from day −10 to day 16. (D) Day 18 LLC lung surface tumor nodule counts in mice with different *Klebsiella* exposures or *Klebsiella* pre-infections, administered *Streptococcus pneumoniae* QBSPN, or left untreated, from day −10 to day 16. SP = QBSPN; PA = QBPAE. Mean +/− SD shown, n = 4–8 mice per group. *, P < 0.05; **, P < 0.01; ****, P < 0.0001 by 1-way ANOVA followed by Tukey's multiple comparison test (A, C and D) or Student's *t*-test (B).

*Klebsiella* species are a common lung pathogen in humans and can be present in animal facilities, including the animal suppliers (Jackson Laboratories and Envigo) for mice used in the experiments shown in Fig. 1. To determine whether *Klebsiella* exposure is required for QBKPN efficacy, animals were sourced from colonies that had environmental *Klebsiella* species exposure (Jackson Laboratories) or from colonies that excluded prior exposure to *Klebsiella* (Taconic excluded flora mice). In contrast to the QBKPN anti-tumor efficacy observed in mice obtained from *Klebsiella* positive colonies, QBKPN administration failed to reduce tumor burden in *Klebsiella-*naïve mice obtained from excluded flora colonies (Fig. 2A). However, exposure of *Klebsiella-*naïve mice derived from excluded flora colonies to live *Klebsiella* by intra-tracheal instillation three weeks prior to QBKPN administration, followed by lung tumor challenge, resulted in QBKPN anti-cancer efficacy (Fig. 2A). Seven days post-infection with live *Klebsiella*, mice tested negative for bacteria-specific genes by PCR (data not shown), demonstrating that overt infection was resolved prior to QBKPN administration, and suggesting that active infection was not the mechanism of anti-cancer efficacy. QBKPN-mediated anti-tumor efficacy was also observed in *Klebsiella-*naïve mice from excluded flora colonies that were infected with *Klebsiella* and rested in micro-isolators in a *Klebsiella*-free barrier facility for at least 6 months prior to QBKPN administration and tumor inoculation (Fig. 2B), discounting the possibility that enhanced anti-tumor efficacy was a consequence of short-term immune activation caused by the infection.

We next assessed whether the anti-tumor efficacy was a consequence of general innate activation by any inactivated bacterial-derived immunotherapy, or whether a species-specific reactivation, dependent upon prior exposure to that bacterial species in the lungs was required. Two additional immunotherapies were generated, one from *Pseudomonas aeruginosa* (QBPAE) and one from *Streptococcus pneumoniae* (QBSPN). Although neither QBSPN nor QBPAE had any efficacy in mice sourced from *Streptococcus-* and *Pseudomonas*-negative colonies, pre-infection of mice with *S. pneumoniae* imparted anti-tumor activity with species matched immunotherapy QBSPN, while pre-infection of mice with *P. aeruginosa* imparted anti-tumour activity with the species matched immunotherapy QBPAE (Fig. 2C). In contrast, we observed only a modest anti-tumor response in *Streptococcus* exposed mice treated with the non-species matched immunotherapy QBPAE and no anti-cancer efficacy was seen in *Pseudomonas-*exposed mice treated with the non-species matched immunotherapy, QBSPN. Additionally, QBSPN had no effect on lung tumor growth (Fig. 2D), regardless of prior *Klebsiella* environmental exposure or *Klebsiella* pre-infection. Thus, optimal induction of lung anti-tumor immunity appears to require prior lung exposure and subsequent treatment with a species-matched immunotherapy.

### QBKPN-mediated anti-tumor efficacy was independent of adaptive immunity

**Figure 3.**
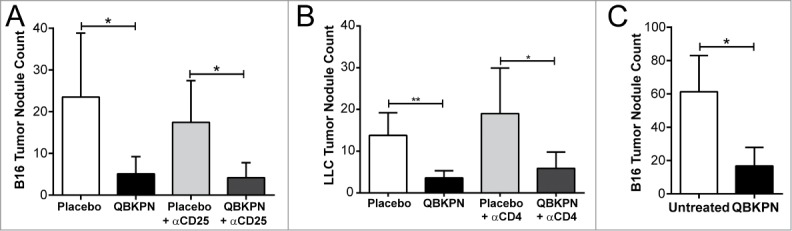
Adaptive immunity was dispensable for QBKPN anti-tumor efficacy. (A) B16F10 left lung surface tumor nodule counts of mice with or without CD25^+^ cell depletion, after administration of placebo or QBKPN from day −10 until euthanasia on day 18. (B) LLC left lung surface tumor nodule counts of mice with or without CD4^+^ cell depletion, after administration of placebo or QBKPN from day −10 until euthanasia on day 16. (C) B16F10 lung surface tumor nodule counts in Rag2 knockout mice administered with QBKPN or left untreated from day −10 until euthanasia on Day 14. Mean +/− SD shown, n = 4–5 mice per group. *, P < 0.05; **, P < 0.01 by Student's *t*-test.

The above observations suggest subcutaneous administration of inactivated bacterial-derived immunotherapies from species previously infecting the lungs, activates a form of immunological memory that leads to anti-tumor efficacy in the lungs. As immunological memory has historically been presumed to require adaptive immunity, we assessed the need for adaptive immunity in our models. Initially, CD25 expressing cells, which includes activated T cells, were depleted (Supplemental Fig. 1 A). Interestingly, the depletion of CD25 expressing cells did not affect the anti-tumor efficacy of QBKPN (Fig. 3A). Next, CD4-expressing cells, which are integral components of adaptive anti-cancer efficacy,^20^ were depleted. Antibody-mediated depletion of CD4 cells (Supplemental Figure 1B) had no effect on QBKPN-mediated anti-tumor efficacy (Fig. 3B). Finally, to exhaustively rule out the reliance on an adaptive immune response for QBKPN efficacy, we tested whether QBKPN administration reduced tumor burden in RAG2 knockout mice which were sourced from colonies that tested positive for *Klebsiella* exposure. QBKPN administration reduced tumor burden in RAG2 knockout mice, and this response was comparable to immune-intact mice (Fig. 3C). Taken together, these findings demonstrate that adaptive immunity was not essential for QBKPN mediated lung anti-tumor efficacy.

### QBKPN administration stimulated a systemic innate immune response leading to innate immune cell increases in the lungs

**Figure 4.**
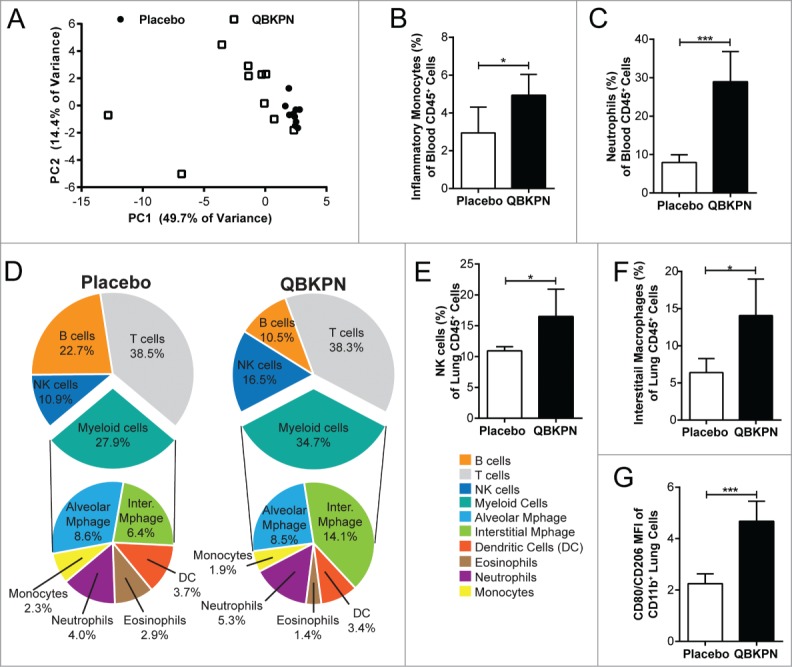
QBKPN stimulated an acute-like immune response, leading to innate cell recruitment into the lungs. (A) Principal component analysis of 31 cytokines/chemokines five hours after a single dose of placebo (circles) or QBKPN (squares) in the serum of mice. The first two principle components (PC) are shown. (B) Percentage of inflammatory monocytes (CD11b^+^CD115^+^Ly6C^HI^ CCR2^+^) and (C) neutrophils (Ly6G^+^) as a percentage of CD45^+^ cells in the blood five hours after a single dose of placebo or QBKPN. (D) Proportion of immune cells in the lungs of mice in a B16F10 lung cancer model, administered placebo or QBKPN from day −10 until euthanasia at day 17. Upper pie charts show the proportion of B cells, T cells, NK cells and myeloid cells. Lower pie charts show the proportion of different myeloid cells. All percentages reflect the percent of CD45^+^ defined cells,^56^ with NK cells (E) and interstitial macrophages (F) as a percentage of CD45^+^ defined cells individually shown. (G) Mean fluorescent intensity (MFI) ratio of CD80/CD206 of the CD11b^+^ lung cells (excluding neutrophils), in a B16F10 lung cancer model, administered placebo or QBKPN from day −10 until euthanasia at day 17. Mean +/− SD shown, n = 5–10 mice per group. *, P < 0.05; ***, P< 0.005 by Student's *t*-test.

Given the demonstrated lack of requirement for adaptive immunity, we investigated the innate immune mechanisms that could drive QBKPN-mediated anti-tumor efficacy. The initial immune response triggered by QBKPN treatment was assessed by measuring changes in serum cytokine levels. Five hours following the first subcutaneous dose, QBKPN caused a large increase in many inflammatory cytokines, including granulocyte-CSF (G-CSF), GM-CSF, IL-1β, IL-2, IL-3, IL-6, IL-7, IL-12(p40), IL-12(p70), IL-13, IL-15, chemokine (C-X-C motif) ligand 1 (CXCL1), macrophage-CSF (M-CSF), and TNFα (Supplemental Table 1). Overall, QBKPN treatment at 5 hours after a first subcutaneous injection caused a clear change in the chemokine/cytokine profile in the serum, as illustrated in the principal component analysis of 31 measured chemokines/cytokines (Fig. 4A).

In parallel to these soluble immune mediators, QBKPN induced an expansion in the percentage of circulating neutrophils and inflammatory monocytes (CD11b^+^CD115^+^ Ly6C^HI^CCR2^+^) within 5 hours of a single QBKPN subcutaneous injection (Fig. 4B, C). This increase in the proportion of myeloid cell populations in blood was mirrored by a trend for a decrease in the proportion of circulating B lymphocytes (QBKPN = 36.3%; Placebo = 32.8%; P = 0.12) and T lymphocytes (QBKPN = 45.1%; Placebo 41.0%; P = 0.16; data not shown).

Next, to determine whether the systemic activation and increased percentages of myeloid cells in the blood was reflected in the lungs, changes in the lung immune profile induced by QBKPN administration were investigated using the B16F10 lung cancer model. At day 17 post-tumor challenge, QBKPN administration resulted in an overall increase in CD45^+^ cells (data not shown) in the lungs and a noticeably different immune cell profile compared to placebo (Fig. 4D). Within immune cells in the lungs, QBKPN administration increased the percentage of NK cells (Fig. 4E) and interstitial macrophages (Fig. 4F), and was associated with a trend towards a greater proportion of neutrophils. QBKPN administration decreased the percentage of eosinophils and B cells in the lungs, while not effecting the total proportion of T cells, alveolar macrophages, monocytes and dendritic cells.

The activation state of macrophages in the lungs has been linked to differential prognosis in lung cancer,^21^ with M2 macrophages (expressing CD206) being more permissive for cancer growth and M1 macrophages (expressing CD80) being generally protective against cancer.^22^ To assess the phenotype of macrophages in the lung after QBKPN administration, we assessed these cellular markers (CD206, CD80) on macrophages (demarked by CD11b expression) from total lung homogenates in mice bearing B16F10 pulmonary tumors. At day 17 post-tumor inoculation, QBKPN treatment increased the ratio of CD80/CD206 expression on lung macrophages (Fig. 4G), demonstrating an increase in the percentage of M1 lung macrophages. To confirm this phenotypic shift, the gene expression of classical markers of M1 (nitric oxide synthase, *iNOS*), and M2, (arginase, *Arg1*) macrophages were assayed in lung homogenates, which showed an increase in *iNOS*, but not in *Arg1* expression in lung tissue (Supplemental Figure 2 A-B), confirming the M1 polarization of lung macrophages with QBKPN administration.

### Anti-tumor efficacy of QBKPN was mediated through NK cells

**Figure 5.**
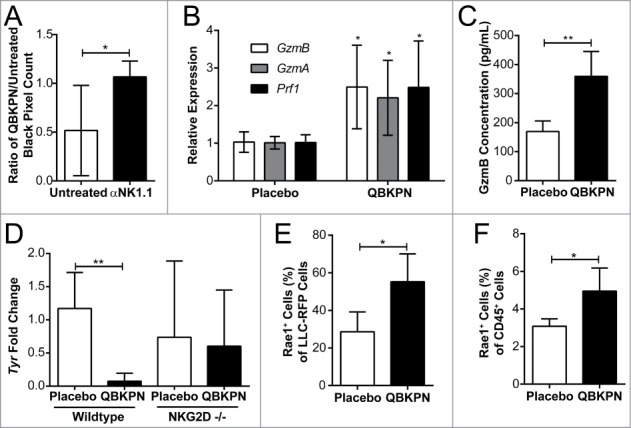
QBKPN anti-tumor efficacy required NK cells and the NKG2D pathway. (A) Mice were treated with anti-NK1.1 antibody or left untreated. The ratio of black pixels (surface B16F10 melanoma tumors) for mice administered with QBKPN to mice untreated from day −10 until euthanasia at day 14. (B) Quantitative RT-PCR of Granzyme B (*GzmB*), Granzyme A (*GzmA*) and Perforin 1 (*Pfr1*) in the lungs of mice administered placebo or QBKPN from day −10 until euthanasia at day 17, in the B16F10 model. (C) Granzyme B protein level in the lungs of mice administered placebo or QBKPN from day −10 until euthanasia at day 17, in the B16F10 lung cancer model. (D) Tumor levels in the lungs of wildtype and NKG2D^−/−^ mice administered QBKPN or placebo from day −10 until euthanasia at day 11, in the B16F10 lung cancer model, measured by quantitative RT-PCR of *Tyr*. (E) Rae1^+^ cells as a percentage of red fluorescent protein (RFP) tagged LLC cells in the lungs of mice administered placebo or QBKPN from day −10 until euthanasia at day 15, in the LLC lung cancer model. (G) Rae1^+^ cells as a percentage of CD45^+^ cells in the lungs of mice administered QBKPN or placebo from day −10 until day 17, in the B16F10 lung cancer model. Mean +/− SD shown. N = 5 mice per group. *, P < 0.05; **, P < 0.01 by Student's *t*-test.

Given that there is crosstalk and cooperative action between macrophages and NK cells for optimal innate anti-cancer immunity,^23^ the role of NK cells in controlling tumor burden in the context of QBKPN administration was assessed using a NK cell depleting antibody.^24^ The efficiency of antibody-mediated depletion of NK cells in the spleen and lungs was verified using flow cytometry (Supplemental Figure 1C, D). NK cell depleted mice had increased tumor engraftment in the lungs. NK cell depletion abrogated the anti-tumor efficacy of QBKPN (Fig. 5A), underscoring the essential contribution of NK-mediated cytotoxicity in QBKPN's anti-tumor efficacy.

NK cells, like cytotoxic T cells, target malignant cells using cytotoxic granules packed with granzymes and perforin.^25^ In comparison to placebo, QBKPN administration increased granzyme B (GzmB), granzyme A (GzmA), and perforin (Prf1) expression in the lungs (Fig. 5B) of B16F10 tumor-bearing mice, which were associated with a decrease in tumor burden. Granzyme B protein levels were subsequently assayed by ELISA and, in corroboration of the gene expression data, were increased in the lungs of QBKPN-administered tumor-bearing mice relative to placebo (Fig. 5C). These data suggest that QBKPN administration enhances NK effector function, contributing to anti-tumor efficacy in the lungs.

Natural killer group 2D (NKG2D), an activating transmembrane receptor belonging to the CD94 family of C-type lectins, is expressed primarily on NK cells and cytotoxic T cells. NKG2D recognizes stress-associated molecules (collectively called NKG2D ligands) that are upregulated on cells upon infection and/or malignant transformation.^26^ It was hypothesized that QBKPN administration may induce or enhance NK cell-mediated anti-tumor efficacy through the NKG2D pathway. In NKG2D knockout mice, QBKPN administration failed to reduce lung tumor burden relative to placebo-treated mice (Fig. 5D), confirming the requirement of NK cell-mediated anti-tumor efficacy through the NKG2D pathway. Toll-like receptor signaling enhances expression of NKG2D ligands in multiple cell types.^27^ Therefore, we assessed whether QBKPN administration altered NKG2D ligand expression on immune and cancer cells. QBKPN administration caused an increase in lung expression of ribonucleic acid export 1 (RAE1), an NKG2D ligand. This increased expression of RAE1 was observed on both cancer cells and CD45^+^ immune cells (Fig. 5E, F). Collectively, these data underscore the importance of the NKG2D pathway and NK cells in QBKPN-mediated anti-tumor efficacy against lung tumors.

### QBKPN exploratory clinical study in patients with NSCLC

Given these promising preclinical results, QBKPN was tested in a small exploratory open-label, single-arm clinical study (NCT02256852) in adult patients who were screened and found to have developed primary pre-invasive or invasive lung adenocarcinomas following surgical resection of stage 1 non-small cell lung cancer (NSCLC). The primary objectives of this trial were to assess the safety, tolerability and compliance to QBKPN treatment. The exploratory endpoints included identifying biomarkers associated with immune response. Tumor imaging by CT scans were carried out prior to QBKPN treatment initiation, near the end of treatment and at a final follow-up >3 months after stopping QBKPN treatment. All patients self-administered QBKPN at home by subcutaneous injection every second day for 12 weeks. Dosing was customized for each patient until a 2.5-5.0 cm diameter circular area of erythema at the injection site on the day following administration was attained, indicating a local immune response at the injection site. A total of 6 patients were enrolled, all female, with a mean age of 66 years (65–70 years). The tumor node metastasis (TNM) classification of malignant tumors (size of tumor; regional lymph node involvement; distant metastasis) at time of study enrolment for all patients was T1N0M0. A baseline demographic summary is provided in Supplemental Table 2.

### QBKPN treatment was well tolerated and caused no serious adverse events in patients with NSCLC

No serious adverse events were reported in the study. Of the adverse events reported, 84.5% were intensity grade 1 (mild) with no adverse effect being higher than intensity grade 2 (moderate). Collectively, 66% of the adverse events were considered unrelated to QBKPN treatment. Five of the six patients reported either grade 1 fatigue or grade 1 tiredness (or both), which resolved entirely without any intervention. Two patients had grade 1 low hemoglobin during treatment. A decrease in hemoglobin was observed in all patients over the course of treatment, but none fell outside of the normal range. Clinical chemistry, hematology, and urinalysis did not identify any additional adverse changes. A summary of adverse events is provided in Supplemental Table 3.

Tolerability and compliance for QBKPN treatment was high. Five of the six patients (83%) completed the full 12 weeks of dosing. The one patient who did not complete 12 weeks of treatment missed the last five planned doses due to a respiratory illness that included cough and phlegm.

### Lung tumor growth was stable and immune changes in the blood were identified with QBKPN treatment

Lung tumor size was assessed utilizing serial computed tomography (CT) evaluations (Table 1). In the pre-QBKPN treatment period (up to 84 months prior to study enrollment), 5 of the 6 patients had experienced increase in lung tumor burden. During the 12 weeks of QBKPN treatment, all patients had stable tumor burden. Over a post-treatment observation period of between 5 to 12 months, 3 of the patients had increases in tumor burden, while, in the other 3 patients, tumor size remained stable.

**Figure 6.**
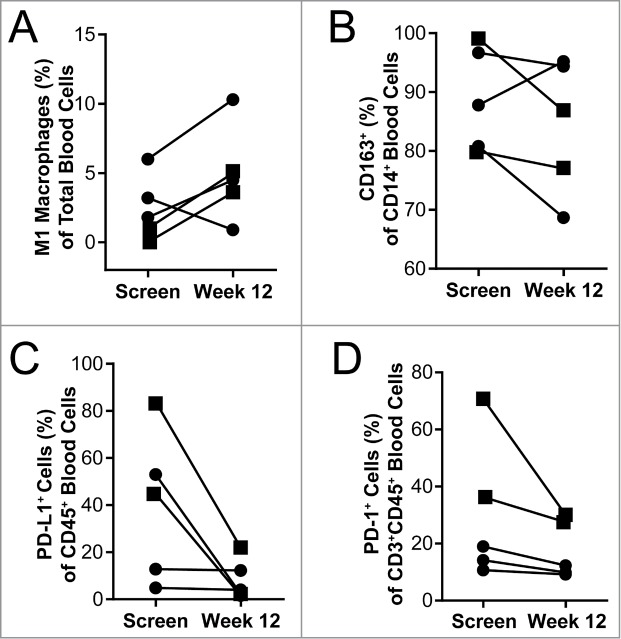
Blood immunology was changed in patients after 12 weeks of QBKPN treatment. (A) M1 macrophages (CD45^+^CD14^+^HLA-DR^+^CD86^+^) as a percentage of all cells in the blood of patients at screening and after 12 weeks of QBKPN treatment. (B) M2 macrophage marker expressing (CD163^+^) cells as a percentage of all CD14^+^ cells in the blood at screening and after 12 weeks of QBKPN treatment. (C) PD-L1^+^ cells as a percentage of all blood immune cells (CD45^+^) at screening and after 12 weeks of QBKPN treatment. (D) PD-1^+^ cells as a percentage of CD3^+^CD45^+^ cells in the blood at screening and after 12 weeks of QBKPN treatment. ▪, Neoplastic tumor bearing patients; •, pre-neoplastic tumor bearing patients.

As indicated above, in mouse models, QBKPN administration altered the monocyte/macrophage profile towards M1 polarization. To assess this in the clinical setting, we measured M1 and M2 monocytes/macrophages in the peripheral blood at screening and after QBKPN treatment (12 weeks) for the 5 patients who had a week 12 blood draw. One patient did not have a useable peripheral blood draw at screening and therefore a blood draw on day 4 post first treatment was used as the baseline value. An increase in the percentage of M1 monocyte/macrophages, as defined as CD45^+^CD14^+^HLA-DR^+^CD86^+^ cells, was observed in 4 of the 5 patients during QBKPN treatment. A decrease in the percentage of CD14^+^ macrophages expressing the M2 marker (CD163) was seen in 4 of the 5 patients (Fig. 6 A,B).

Immune suppression was assessed in blood samples of trial participants by measuring the percentage of PD-L1 expressing CD45^+^ cells and PD-1 expressing CD3^+^CD45^+^ cells, at baseline and at week 12 of QBKPN treatment. There was a trend for a reduction in PD-L1 and PD-1 positive cells, over the course of treatment, in all five patients for whom a week 12 sample was available (Fig. 6C, D). Three patients had higher percentages of PD-L1 expressing CD45^+^ cells at baseline and these patients had a trend for a larger decrease in PD-L1^+^ cells (average decrease of 52%) with QBKPN treatment, compared to the two patients had low levels of PD-L1 expressing CD45^+^ cells at baseline (average decrease of 0.7%). A similar trend was seen for the PD-1 positive cells with QBKPN treatment. Based on radiological imaging, two patients were classified as having neoplastic disease, whereas four patients were classified as having pre-neoplastic lesions. Of note, the two patients diagnosed as having neoplastic disease had a trend for elevated PD-L1 and PD-1 expressing cells at baseline, compared to the patients diagnosed as having pre-neoplastic disease.

## Discussion

Induction of innate immunity through bacterial-derived immunotherapies is a potentially attractive strategy for cancer therapy, but there has been limited success in translating this approach into safe, reliable and efficacious immunotherapies.^1,5^ Standard treatment options for progressive lung cancer in patients who are ineligible for surgery include palliative radiation and chemotherapy. There has recently been a surge of interest and success in immunotherapeutic approaches for lung cancer, primarily with checkpoint inhibitors, including PD-1/PD-L1 pathway checkpoint blockades. However, the majority of this interest in immunotherapies has centered on activating and/or utilizing the adaptive arm of the immune system, rather than activating innate immune function. As lung cancer remains a leading cause of cancer-related death with a five-year survival rate of only 18%,^28^ there still remains an urgent need for novel, safe and non-toxic treatments. In this proof-of-principle study, a bacterial-derived immunomodulator of the innate immune system, QBKPN, was tested in mouse models of lung cancer and in a small exploratory study in NSCLC patients. QBKPN administration demonstrated anti-cancer efficacy by reducing lung tumor burden and improving survival in experimental models of metastatic lung cancer, through the activation of multiple anti-cancer immune pathways in the lungs. In the small exploratory study in NSCLC patients, short-term safety and tolerability to QBKPN treatment was demonstrated, along with high compliance to the treatment regimen, no identified serious adverse events, altered blood immune environment, and stable disease during the three months of treatment. Collectively, this data suggests that using QBKPN to harness an innate anti-cancer immune response in the lungs is an attractive novel approach that warrants further investigation.

The association between acute infection and spontaneous cancer remission has been well-documented throughout history and although the mechanism is not fully elucidated, acute immune activation and recruitment of innate immune cells are hypothesized to be critical for this phenomenon.^1,4^ This concept is illustrated in the use of BCG, a live bacterial treatment for bladder cancer that has been applied clinically for decades and is proposed to work through multiple innate immune mechanisms.^4^ BCG has been shown to induce an immune response in bladder tissue that includes increases in NK cells, neutrophils and macrophages, and release of many of the cytokines demonstrated to be increased with QBKPN treatment, including IL-1, Il-2, IL-6, IL-12, GM-CSF and TNFα.^4,29^ Systemically, QBKPN treatment simulates an acute immune response to bacterial infection, including increases in monocytes and neutrophils and an acute pro-inflammatory cytokine response.^30^ However, a circulatory acute inflammatory response alone is not optimal for anti-cancer efficacy for tumors situated in a specific organ, as evidenced by the limited clinical anti-cancer efficacy of TLR agonist therapies that create a broad systemic response.^31^ Immune stimulation in the cancer tumor and/or the specific organ environment is believed to be critically important for anti-cancer efficacy. For example, according to Coley's clinical observations, Coley's Toxin was most beneficial when delivered directly at and around the tumor site.^3^ Similarly, BCG is introduced directly into the bladder as intravesical therapy for bladder cancer,^4^ and experimental live bacterial vaccines require colonization of the tumor environment.^5^ While QBKPN is administered subcutaneously, and thus, at a distance from the site of malignancy, QBKPN administration induced an acute inflammatory response in mice lungs, including innate immune cell recruitment with large increases in the proportion of interstitial macrophages and NK cells. Similar results were demonstrated with QBKPN treatment in disease-free mice and mice with smoke-induced lung inflammation.^16^ This data collectively shows that subcutaneous administration of QBKPN resulted in increased circulatory innate immune cells, but also, and more importantly, culminated in increased innate immune cells in the lungs.

Within the organ containing the tumor, there is a dynamic interaction between the innate immune response and the tumor. In this context, macrophage polarization, not just the absolute number of macrophages, plays an important role in determining outcome.^21,22^ Macrophages can have a diverse range of influence on the tumor depending on their M1/M2 polarization. On the extremes of the M1/M2 spectrum, tumor associated macrophages (M2) increase tumor invasion and promote tumor growth, while M1 macrophages are an important component of an anti-cancer immune response.^21,22^ For many cancers, including lung cancer, higher levels of M1 macrophages in the cancer environment are associated with a more favorable prognosis.^32^ This correlation has resulted in the search for cancer therapeutic strategies that manipulate macrophage in the tumor environment, including cytokine therapies in mice^33^ and humans.^34^ QBKPN, which increased the number and M1 polarization of lung macrophages in mice and systemically in patients, may be an attractive approach to shift tumor associated macrophage dominance to an M1 profile while avoiding toxicity issues presented with other macrophage directed therapies, including cytokine therapies.^35^ The precise contribution of M1 macrophages in QBKPN anti-cancer efficacy has not been fully determined, however, an M2 to M1 macrophage shift can impact multiple potential mechanisms leading to anti-cancer efficacy, including reduction of both angiogenesis and immune suppression, and activation of other anti-cancer effector cells, including NK cells.^23,36^

NK mediated cytotoxicity is a central mechanism of anti-cancer immunity.^25^ QBKPN administration increased NK cells in the lungs of lung-tumor bearing mice, which were shown to be required for QBKPN anti-cancer efficacy. Multiple pathways lead to NK cell activation and anti-cancer cytotoxicity, including cytokine stimulation, NKG2D activation, and stimulation by other cells types,^25,37^ all of which were activated by QBKPN administration. QBKPN administration increased multiple cytokines important for NK cell activation, proliferation and survival, including IL-2, IL-12 and IL-15.^37^ However, cytokines work in concert with additional pathways, including the NKG2D pathway, to optimally activate NK cells.^37,38^ NKG2D is an activating receptor located on NK cells and other cells, which recognizes a diverse array of ligands important in the stress response.^26^ QBKPN administration resulted in an increase in NKG2D ligand (RAE1) positive immune cells and cancer cells in mouse lungs, suggesting an increased stress response in the tumor.^26^ This is consistent with the observation that bacterial and TLR stimulation increases expression of NKG2D ligands on immune cells^27,39,40^ and cancer cells.^41^ In addition to NK cell activation by cytokine stimulation and the NKG2D pathway, interaction with other immune cells can contribute to NK cell activation.^42^ For example, co-ordination between NK cells and macrophages, including through the NKG2D pathway,^27,39^ leads to NK cell activation and, ultimately, cancer cytotoxicity. Collectively, these data suggest that QBKPN administration simultaneously stimulates multiple pathways for NK cell activation, ultimately leading to NKG2D-mediated cancer cytotoxicity.

QBKPN treatment in patients was observed to decrease the number of both PD-1 and PD-L1 expressing circulating immune cells. NSCLC patients often have elevated expression of the PD-1/PD-L1 check-point pathway in lung tumors,^43,44^ which contributes to the immune suppression associated with this disease. Blocking checkpoint pathways has been extensively investigated as a therapeutic strategy in cancer, leading to the recent approval of PD-1 and PD-L1 blocking antibodies for the treatment of NSCLC.^45^ In the clinical trial, PD-L1 status was not assessed on cancer cells; but PD-1 and PD-L1 were assessed on circulating immune cells, demonstrating a reduction of both PD-1 and PD-L1 cells in all 5 assessed patients. Although not confirmed with histological evidence, two patients had tumors classified as neoplastic based on CT scan imaging, and these patients had higher levels of both PD-L1 and PD-1 cells. QBKPN treatment resulted in a large drop in PD-1 and PD-L1 cells in these two patients. In contrast, the three patients with pre-neoplastic tumors, based on CT scans, had, in general, lower percentage of PD-L1 and PD-1 cells at baseline and QBKPN treatment had no obvious effect on PD-L1 and PD-1 cell levels in these patients. These trends may suggest that QBKPN treatment may shift immune function towards an anti-cancer environment without forcing an imbalance of immune homeostasis, which is important given the growing recognition of potential immune-related adverse events related to the use of checkpoint inhibitors.^46^ Additional studies will be required to determine if these observations and trends hold in a larger set of patients.

QBKPN anti-cancer efficacy was independent of adaptive immunity but required species-specific past exposure, suggesting an innate memory mechanism. Multiple innate immune cells have been demonstrated to have a memory phenotype, including monocytes, NK cells and innate lymphoid cells.^47^ In contrast to the highly specific nature of B and T cell memory, innate memory is primarily considered to be a broad non-antigen-specific response.^47^ However, although NK cells can provide nonspecific protection to stimuli,^48^ NK cells have also been demonstrated to have stimulus-specific memory, including to haptens and viruses.^49,50^ NK cells can develop memory for bacteria,^51^ but the specificity of this innate memory has not been determined. In our studies, immunotherapies produced from *Klebsiella, S. pneumoniae*, and *P. aeruginosa* all required species-specific pre-exposure for anti-cancer efficacy in mice, suggesting that innate immune memory in this context is species specific. These results suggest that both gram positive and gram negative bacteria could be used for production of the immunotherapies; thus, the response is not solely dependent on lipopolysaccharide stimulation.

To continuously re-stimulate an innate immune response, QBKPN was administered every second day. The innate immune response to acute bacterial stimulation is rapid and short-lived, resolving within hours to days.^52^ This hypothesis with respect to the importance of frequent repeated dosing to continuously stimulate an anti-cancer innate immune response is consistent with the clinical observations by Dr. Coley.^3^ Every second day dosing of QBKPN was well tolerated in the clinic with no identified adverse events above level 2, which is consistent with the good safety profile of inactivated bacterial products.^53^ Subcutaneous administration of QBKPN enables self-administration in the home setting, a substantial advantage over cancer therapies that require administration by a healthcare provider in a clinical setting. Although the treatment period (12 weeks) in the exploratory clinical trial was insufficient to document clinical efficacy, it was promising that tumor burden, as measured by CT scan, remained stable during the 12-week QBKPN treatment period in all patients.

Collectively, these finding demonstrate that a novel immunotherapy, QBKPN, is an effective activator of the innate immune response in the lungs, leading to anti-cancer efficacy. Subcutaneous QBKPN administration harnessed multiple important anti-cancer immune pathways by mimicking an acute infection, resulting in increased innate immune cells in the lungs, M1 macrophage polarization, and activation of NK cells. QBKPN efficacy required species-specific pre-exposure but was independent of the adaptive immune system. In the clinic, QBKPN treatment was well-tolerated with a good safety profile and immunological response suggestive of immune stimulation. Additional clinical studies are planned to assess anti-cancer efficacy, including impact on survival, using this innovative immunotherapy approach.

## Materials and methods

### Animals

Female mice C57BL/6 (aged 6–10 weeks) were sourced from multiple suppliers. C57BL/6 J mice sourced from Jackson Laboratories (Bar Harbor, ME, USA) and Envigo (Livermore, CA, USA) came from facilities that had previously tested positive for *Klebsiella* species exposure during routine screening. Mice (C57BL/6NTac Excluded Flora [IBU14]) sourced from Taconic (Germantown, MD, USA) came from facilities that were certified negative for *Klebsiella* species exposure. NKG2D knockout mice (B6.Cg-Kirk1<tm1Djr>/J) and RAG-2-deficient mice (B6.Cg-Rag2<tm1.1Cgn>/J) were purchased from Jackson Laboratories. Mice were acclimatized and housed for at least one additional week prior to the experimental studies and were contained in environmentally controlled conditions with a 12:12 hour light/dark cycle for the duration of the study. For the infection and exposure studies, mice were housed in microisolator units under pathogen-excluding barrier conditions to limit access and transfer of micro-organisms. All protocols completed at Dartmouth College were in accordance with the Dartmouth Institutional Animal Care and Use Committee guidelines. All protocols completed at Qu Biologics were in accordance with the Canadian Council on Animal Care's policies and guidelines.

### Bacteria-based intervention strategy

The bacterial-derived investigational therapeutic, QBKPN, (Qu Biologics Inc., Vancouver, BC, Canada) is a proprietary immunomodulator consisting of all major macromolecules of an inactivated pathogenic *Klebsiella* strain originally derived from a patient isolate, as described.^15,16^ For standard studies, 30 μL of QBKPN or a placebo vehicle control (physiological saline, with or without 0.45% phenol) was subcutaneously injected in a rotating manner every other day into skin folds on the animal's lower right abdomen, upper right chest, upper left chest, and lower right abdomen. For intervention studies, QBKPN was administered every second day starting 10 days before tumor cell inoculation and every second day after tumor cell inoculation until mice were euthanized. QBKPN and placebo were not given on the day of tumor inoculation. For the treatment studies, QBKPN injections were started 24 hours after the tumor cell inoculation and continued every second day until the mice were euthanized. Two additional proprietary bacterial immunotherapies, QBSPN and QBPAE were also produced. QBSPN was produced from *Streptococcus pneumoniae* and QBPAE was produced from *Pseudomonas aeruginosa*, using a similar method to the production of QBKPN.

#### Tumor inoculation and evaluation

B16F10 melanoma cells (ATCC CLR-6475, Manassas, VA, USA), Lewis Lung Carcinoma (LLC) cells (ATCC CRL-1642) and red fluorescent protein (RFP)-tagged LLC cells (AntiCancer Inc.) were obtained and cultured in RPMI media supplemented with 10% FBS. For tumor studies, cells were harvested and resuspended in PBS, and then injected intravenously by tail vein injection at 2 × 10^5^ cells for the B16F10 cells and 1 × 10^5^ to 4 × 10^5^ cells for the LLC cells in 100 uL PBS. Tumor burden was assessed by enumerating the visible surface metastatic lung lesions; LLC-infiltrated lungs were stained with Bouin's solution (Sigma-Aldrich, St Louis, MO, USA) to provide contrast.^54^ Relative levels of B16F10 burden were also evaluated using established PCR-based assessment for the melanocyte differentiation gene product Tyrosinase (*Tyr*),^19^ which is expressed by the melanoma cells but not by the lung tissue. For the NK depletion study, due to higher seeding on tumors compared to untreated mice, the number of black pixels (representing the B16F10 melanomas) were counting with ImageJ v1.50.^55^ Data is shown as the ratio of black pixels for mice administered with QBKPN to the average black pixels for mice untreated, for both the untreated group and the αNK1.1 antibody treated group.

#### Bacterial infection

In some studies, mice were pre-exposed to specific bacteria prior to treatment with the bacterial-derived immunotherapies. Bacteria, which included the parental *Klebsiella* strain that was used to generate QBKPN, *Streptococcus pneumoniae* strain P1121 (gift of Dr. George O'Toole), or *Pseudomonas aeruginosa* stain PA14 (gift of Dr. Brent Berwin), were grown overnight in LB broth at 36 °C. The concentration of bacteria in broth was determined by measuring the absorbance at 600 nm and plotting the optical density (OD) on a standard curve of known colony forming units (CFU) values. Bacteria were washed and resuspended in PBS at 1 × 10^6^ CFU per 50 μL. Animals were infected intratracheally with 1 × 10^6^ CFU of *Klebsiella*, 1 × 10^5^ CFU of *S. pneumoniae*, or 2.5 × 10^5^ CFU of *P. aeruginosa*, under isofluorane anesthesia, and allowed to rest for three weeks prior to further treatment or tumor challenge. Efficiency of the infection protocol and subsequent clearance of infection was confirmed by the presence and absence, respectively, of specific bacterial gene products in lungs of parallel cohort animals by PCR (data not shown).

#### Antibody depletion and blocking studies

CD4 cells were depleted using 100 μg GK1.5 (eBioscience/Thermo-Fisher, Carlsbad, CA, USA) by intraperitoneal injection and administered 13, 11 and 4 days before tumor inoculation, plus 3 and 10 days post tumor inoculation. The low affinity receptor for IL-2, CD25 (IL-2Rα), was targeted to deplete T cells and B cells activated by QBKPN using intraperitoneal injection of 150 μg of α-mCD25/IL2Rα (PC61, Ablab, Vancouver, BC, Canada). Anti-mCD25/IL2Rα was administered by intraperitoneal injection on day 13 and day 6 before tumor inoculation, and then again on day 4 post-tumor inoculation. NK cells were depleted using 50 μg PK136 (BioXCell, Lebanon, NH, USA) intraperitoneal, every four days starting one day before tumor inoculation.

#### Immune mediator profiling of serum samples

Serum immune factors were analyzed by multiplex technology (performed by Eve Technologies, Calgary, AB, Canada) using a 31 cytokine/chemokine/growth factor kit (Millipore, St Charles, MO, USA). The assay was run on a Bio-Plex™ 200 system (Bio-Rad Laboratories, Inc., Hercules, CA, USA).

#### Antibodies and flow cytometry for murine studies

Anti-mouse antibodies CD16/32, CD45 (30–F11), CD4 (GK1.5), CD8 (53–6.7), NK1.1 (PK136), Ly6G (1A8), Ly6 C (HK1.4), F4/80 (BM8), CD11b (M1/70), MHC class II (M5/114.15.2), Rae1 (CX1), CD64 (X54-5/7.1), CD24 (M1/69) and CD11 c (N418) were sourced from BioLegend (San Diego, CA, USA) and eBioscience (Waltham, Massachusetts, USA). Red blood cells were removed using Red Blood Cell Lysis Buffer (Biolegend). Lungs were harvested and dissociated into a single cell suspension using the Milltenyi Biotec (San Diego, CA, USA) mouse lung dissociation kit and gentleMACS tissue dissociator. Cell viability was assessed using the live/dead fixable violet cell stain kit (ThermoFisher Scientific). Flow cytometry was performed on a MacsQuant 10 (Milltenyi Biotec) or CytoFlex (Beckman-Coulter, Indianapolis, IN, USA). Data were analyzed using FlowJo software (version 9.3.3 or 10.2, Ashland, OR, USA). Lung immune cell profiles were created using a protocol modified from Yu et al.^56^

#### Gene expression

The right lung post-caval lobe was homogenized by a TissueLyser LT (Qiagen, Toronto, ON, Canada). RNA was isolated using the PureLink RNA Mini Kit (Life Technologies, Carlsbad, CA, USA) and reverse transcribed into cDNA using the iScript cDNA Synthesis Kit (Bio-Rad). Gene expression was quantified by quantitative RT-PCR using a StepOnePlus RT-PCR machine (Applied Biosystems, Foster City, CA, USA), TaqMan Fast Advanced Master Mix (Applied Biosystems) and TaqMan probes (Applied Biosystems) for granzyme A (Mm01304452_m1), granzyme B (Mm00442837_m1), perforin (Mm00812512_m1) and Tyrosinase (Mn00495817_m1). All genes were normalized against GAPDH (Mm99999915_g1).

#### Granzyme B protein analysis in lung samples

Right lung caudal lobes were homogenized by TissueLyser LT and processed as per the manufacturer's protocol (Qiagen). Granzyme B protein was quantified by ELISA according to the manufacturer's protocol (eBioscience).

#### Clinical trial patients

Six adult patients with primary pre-invasive or invasive adenocarcinoma following prior surgical resection of stage 1 non-small cell lung cancer (NSCLC) with curative intent were recruited (NCT02256852). Patients with extra-thoracic lung cancer progression, transient lung nodules that resolved or became significantly smaller within three months prior to the study, any active malignancies, any past radiation or systemic therapies to treat prior malignancies or current diagnosis of lung cancer, oral prednisone therapy in excess of 10 mg per day, and any immunosuppressive disorder were excluded from the study. Radiological evidence of lung cancer was defined as persistent or growing non-solid (ground glass) nodules greater that 10 mm or part solid (semi-solid) nodules. Radiological evidence of lung cancer was required to be confirmed in consecutive CT scans at least three months apart. Histological confirmation of malignancy was not completed. Patients were classified as neoplastic or pre-neoplastic based on CT scans. Neoplastic patients were defined as having micro-invasive or invasive adenocarcinomas as determined by semi-solid/solid nodules on CT. Pre-neoplastic patients had atypical adenomatous hyperplasia and adenocarcinoma-in-situs, as determined by ground glass nodule opacities on CT scan.

#### Clinical study design

This was a single center, open label trial. The study protocol was approved by the research ethics board at the BC Cancer Agency, and written informed consent was obtained from participants prior to screening for eligibility. This trial was conducted according to the tenets of the Declaration of Helsinki. Patients underwent a 28-day screening period, 12 weeks of QBKPN treatments, then 4 weeks of post-treatment follow-up. QBKPN was self-administered by subcutaneous injection every 2^nd^ day for up to 12 weeks. The starting dose was 0.05 mL, and was titrated up or down, until a local skin immune response (LSIR) of 2.5 to 5.0 cm was observed the day after injection. Safety variables included hematology, clinical chemistry, urinalysis, vital signs, physical exam, and assessment of adverse events. Changes in tumor size were assessed by serial CT evaluation.

#### Clinical trial circulating immunology assessment

Peripheral blood samples were collected and analyzed by flow cytometry. Anti-human antibodies included CD45(2D1), CD14(61D3), CD86(B7-2), HLA-DR(LN3), CD206(19.2), CD45(2D1), CD3(OKT3), PD-L1(MIH1) and PD-1(J105). Flow cytometry was run on a BD FACSCalibur (BD Bioscience) and analyzed using FlowJo V10.2. Relative levels of classically activated monocytes/macrophages (commonly referred to as M1) in the blood were determined by looking at the percentage of total CD45^+^ cells that were CD45^+^CD14^+^HLA-DR^+^CD86^+^. Alternatively-activated monocytes/macrophages (M2) levels in the blood were assessed as the percentage of CD14^+^ cells that were CD163 positive and HLA-DR^−^CD86^−^. PD-L1^+^ cells were assessed as a percentage of blood CD45^+^ cells while PD-1^+^ cells were assessed as a percentage of CD45^+^CD3^+^ cells in the blood.

#### Data analysis

GraphPad Prism 6 Software (GraphPad Software, San Diego, CA, USA) was used to perform statistical analyses. Data are expressed as mean ± SD. For comparison between 2 groups, Student's *t*-test was performed. For comparison between multiple groups, one-way ANOVA analysis followed by multiple comparisons using a Sidak post-hoc test was performed. Differences between survival was determined by Log-rank (Mantel-Cox) analysis. Differences were reported as statistically significant when p < 0.05.

## Supplementary Material

supp_data.zip
