# Supplementary material for: Harnessing innate lung anti-cancer effector functions with a novel bacterial-derived immunotherapy
Source: Oncoimmunology. 2017 Nov 27;7(3):e1398875. doi: 10.1080/2162402X.2017.1398875 (PMC5790356; doi:10.1080/2162402X.2017.1398875)
Supplement: supp_data.zip [file koni-07-03-1398875-s001.zip › 2017ONCOIMM0574R-f07-z-4c.docx]

## SUPPLEMENTAL FIGURES


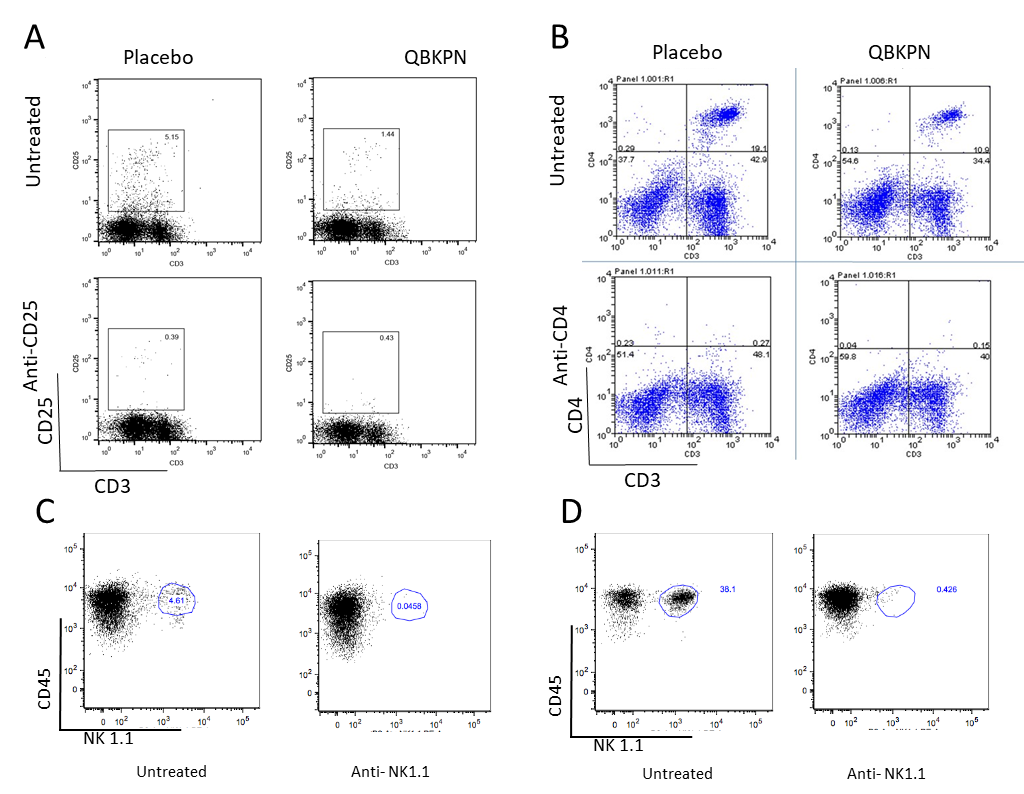


### **Supplemental Figure 1:** Depletion of CD25 cells, CD4 cells and NK cells

(A) Efficacy of CD25 blocking in the spleen of mice administered placebo or QBKPN from day -10 until euthanasia on day 18, in the B16F10 model. (B) Efficacy of CD4 blocking in the lungs of mice administered placebo of QBKPN from day -10 until euthanasia on day 16, in the B16F10 model. Efficacy of NK cell blocking in the spleen (C) or lungs (D) of mice administered untreated of administered anti-NK1.1 antibodies from day -10 until euthanasia on day 14, in the B16F10 model.

### **Supplemental Figure 2:** *iNOS* and *Arg1* expression in the lungs of mice

(A) Relative expression of nitric oxide synthase (*iNOS*) and (B) Arginase 1 (*Arg1*) in the lung of mice administered QBKPN or placebo every second day from day -10 until euthanasia at day 17, in the B16F10 model. N= 10 per group. *, P <0.05.
