# Supplementary material for: Harnessing innate lung anti-cancer effector functions with a novel bacterial-derived immunotherapy
Source: Oncoimmunology. 2017 Nov 27;7(3):e1398875. doi: 10.1080/2162402X.2017.1398875 (PMC5790356; doi:10.1080/2162402X.2017.1398875)
Supplement: supp_data.zip [file koni-07-03-1398875-s001.zip › 2017ONCOIMM0574R-file002.docx]

## SUPPLEMENTAL TABLES

### Supplemental Table 1. Cytokine and chemokine concentrations in serum of placebo and QBKPN-treated animals, five hours after a single dose.

N = 10 mice per group. G-CSF, Granulocyte-CSF; M-CSF, macrophage-CSF; CXCL, chemokine (C-X-C motif) ligand; LIF, leukemia inhibitory factor; CCL, chemokine (C-C motif) ligand; VEGF, vascular endothelial growth factor.

| Analyte | Placebo | | QBKPN | | P value,  Student’s *t*-test |
| --- | --- | --- | --- | --- | --- |
|  | **Average (pg/uL)** | **SD** | **Average (pg/uL)** | **SD** |  |
| Eotaxin | 862.92 | 176.76 | 886.83 | 424.78 | 0.8722 |
| G-CSF | 407.27 | 575.63 | 13479.06 | 15587.63 | 0.0264 |
| GM-CSF | 24.06 | 5.23 | 31.06 | 6.79 | 0.0194 |
| IL-1a | 269.18 | 148.23 | 420.36 | 251.41 | 0.1228 |
| IL-1B | 19.84 | 10.08 | 43.71 | 15.01 | 0.0007 |
| IL-2 | 7.77 | 3.66 | 18.61 | 7.73 | 0.0015 |
| IL-3 | 0.33 | 0.03 | 0.56 | 0.27 | 0.0233 |
| IL-4 | 0.32 | 0.00 | 0.32 | 0.02 | 0.3272 |
| IL-5 | 21.86 | 19.77 | 43.05 | 37.13 | 0.1340 |
| IL-6 | 0.77 | 0.43 | 368.66 | 233.37 | 0.0008 |
| IL-7 | 1.27 | 1.14 | 6.36 | 6.29 | 0.0315 |
| IL-9 | 47.02 | 39.69 | 60.85 | 59.78 | 0.5511 |
| IL-10 | 2.78 | 0.69 | 46.66 | 81.43 | 0.1225 |
| IL-12 (p40) | 1.92 | 2.92 | 23.96 | 27.85 | 0.03340 |
| IL-12 (p70) | 6.33 | 5.78 | 14.95 | 7.91 | 0.0131 |
| IL-13 | 22.87 | 8.49 | 47.95 | 16.60 | 0.0009 |
| IL-15 | 8.64 | 12.32 | 48.85 | 53.53 | 0.0432 |
| IL-17 | 0.72 | 0.57 | 2.56 | 2.59 | 0.0533 |
| CXCL10 | 85.94 | 15.75 | 1010.42 | 1761.29 | 0.1313 |
| CXCL1 | 126.01 | 54.49 | 2204.54 | 2005.23 | 0.0096 |
| LIF | 0.32 | 0.04 | 0.96 | 0.96 | 0.0647 |
| CXCL5 | 11362.84 | 5264.91 | 7836.11 | 9472.57 | 0.3208 |
| CCL2 | 27.54 | 23.62 | 1110.56 | 2562.86 | 0.2143 |
| M-CSF | 12.73 | 4.41 | 21.72 | 9.13 | 0.0149 |
| CXCL9 | 78.02 | 20.52 | 341.26 | 549.05 | 0.1634 |
| CCL3 | 62.34 | 15.15 | 130.52 | 107.82 | 0.0778 |
| CCL4 | 38.21 | 19.05 | 617.19 | 1045.36 | 0.1138 |
| CXCL2 | 184.51 | 45.30 | 235.72 | 67.91 | 0.0651 |
| CCL5 | 23.52 | 9.34 | 137.92 | 234.04 | 0.1567 |
| TNFα | 1.03 | 2.34 | 21.63 | 26.71 | 0.0376 |
| VEGF | 0.81 | 0.18 | 1.17 | 0.49 | 0.0513 |

**Supplemental Table 2. Baseline patient characteristics**

| Characteristic | Result |
| --- | --- |
| Age - (years) |  |
| Mean | 66 |
| Median | 65.5 |
| Range | 65 - 70 |
| Sex - n (%) |  |
| Female | 6 (100) |
| Race - n (%) |  |
| White | 4 (66.7) |
| Asian | 2 (33.3) |
| Pathological TNM Stage at time of initial diagnosis - n (%) |  |
| Stage 1 |  |
| T1N0M0 | 6 (100) |
| Time of Inclusion in Study Since Initial Diagnosis - (years) |  |
| Mean | 4 |
| Median | 4 |
| Range | 2-7 |
| ECOG Performance Status - n (%) |  |
| 0 | 6 (100) |
| 1 | 0 |
| 2 | 0 |

### Supplemental Table 3: Adverse event summary for NSCLC patients treated with QBKPN for 12 weeks.

Number (%) of patients with adverse events by system organ class and preferred term

| **System organ class** |  |
| --- | --- |
| ***Preferred term*** | **Number (%) of patients (n = 6)** |
| Any system organ class | 6 (100.0) |
| Ear and labyrinth disorders | 1 (16.7) |
| *Motion sickness* | 1 (16.7) |
| Gastrointestinal disorders | 2 (33.3) |
| *Nausea* | 1 (16.7) |
| *Paraesthesia oral* | 1 (16.7) |
| General disorders and administration site conditions | 6 (100.0) |
| *Chest discomfort* | 1 (16.7) |
| *Fatigue* | 5 (83.3) |
| *Feeling cold* | 1 (16.7) |
| *Feeling of body temperature change* | 1 (16.7) |
| *Impaired healing* | 1 (16.7) |
| *Influenza like illness* | 1 (16.7) |
| *Injection site mass* | 3 (50.0) |
| *Injection site vesicles* | 1 (16.7) |
| *Pyrexia* | 2 (33.3) |
| *Sensation of foreign body* | 1 (16.7) |
| Infections and Infestations | 2 (33.3) |
| *Nasopharyngitis* | 1 (16.7) |
| *Sinusitis* | 1 (16.7) |
| Injury, poisoning and procedural complications | 2 (33.3) |
| *Arthropod bite* | 1 (16.7) |
| *Chest injury* | 1 (16.7) |
| Investigations | 3 (50.0) |
| *Haemoglobin decreased* | 2 (33.3) |
| *Heart rate increased* | 1 (16.7) |
| *Liver function test abnormal* | 1 (16.7) |
| Musculoskeletal and connective tissue disorders | 2 (33.3) |
| *Muscle spasms* | 1 (16.7) |
| *Tendonitis* | 1 (16.7) |
| Nervous system disorders | 2 (33.3) |
| *Headache* | 2 (33.3) |
| Reproductive system and breast disorders | 1 (16.7) |
| *Breast pain* | 1 (16.7) |
| Respiratory, thoracic and mediastinal disorders | 5 (83.3) |
| *Allergic sinusitis* | 1 (16.7) |
| *Cough* | 3 (50.0) |
| *Dysphonia* | 1 (16.7) |
| *Dyspnoea* | 1 (16.7) |
| *Oropharyngeal pain* | 1 (16.7) |
| *Productive cough* | 4 (66.7) |
| *Rhinorrhoea* | 2 (33.3) |
| *Sinus congestion* | 1 (16.7) |
| *Wheezing* | 1 (16.7) |
| Skin and Subcutaneous tissue disorders | 2 (33.3) |
| *Eczema* | 1 (16.7) |
| *Pruritis* | 1 (16.7) |
| *Rash* | 1 (16.7) |
| *Skin discoloration* | 1 (16.7) |
